# A functional *Notch*–*survivin *gene signature in basal breast cancer

**DOI:** 10.1186/bcr2200

**Published:** 2008-11-24

**Authors:** Connie W Lee, Karl Simin, Qin Liu, Janet Plescia, Minakshi Guha, Ashraf Khan, Chung-Cheng Hsieh, Dario C Altieri

**Affiliations:** 1 Department of Cancer Biology and the Cancer Center, University of Massachusetts Medical School, 364 Plantation Street, Worcester, MA 01605, USA; 2 Department of Medicine, Preventive and Behavioral Medicine, University of Massachusetts Medical School, 364 Plantation Street, Worcester, MA 01605, USA; 3 Department of Pathology, University of Massachusetts Medical School, 364 Plantation Street, Worcester, MA 01605, USA

## Abstract

**Introduction:**

Basal-type, or triple-negative, breast cancer (lacking estrogen receptor, progesterone receptor, and human epidermal growth factor receptor-2 expression) is a high-risk disease for which no molecular therapies are currently available. We studied genetic signatures of basal breast cancer potentially suitable for therapeutic intervention.

**Methods:**

We analyzed protein expression of the Notch-1 intracellular domain and survivin by immunohistochemistry in a series of basal breast cancer patients. A hierarchical clustering and overall survival analysis was carried out on a microarray mRNA database of 232 breast cancer patients. Fifteen published mRNA datasets containing estrogen receptor-negative or estrogen receptor-positive samples were subjected to meta-analysis for co-segregated gene expression. Experiments of plasmid transfection and gene silencing were carried out in estrogen receptor-negative MDA-MB-231 breast cancer cells.

**Results:**

The developmental signaling regulator Notch-1 was highly expressed in breast cancer, compared with normal tissue, and was segregated with basal disease. Higher *Notch-1 *levels correlated with progressively abbreviated overall survival, and with increased expression of *survivin*, a tumor-associated cell death and mitotic regulator implicated in stem cell viability. Analysis of Pearson's correlation coefficient indicated that *Notch-1 *and *survivin *co-segregated in basal breast cancer. Notch-1 stimulation in MDA-MB-231 cells increased survivin expression, whereas silencing Notch reduced survivin levels.

**Conclusions:**

A *Notch-1–survivin *functional gene signature is a hallmark of basal breast cancer, and may contribute to disease pathogenesis. Antagonists of Notch and survivin currently in the clinic may be tested as novel molecular therapy for these recurrence-prone patients.

## Introduction

The introduction of molecular gene signatures in breast cancer provides important prognostic and predictive information [1-3], and holds promise for individualized molecular therapy of these patients [4]. Certain subtypes of breast cancer, however, continue to pose therapeutic challenges [4]. For example, basal breast cancer is a myoepithelial disease variant characterized by high histologic grade [5], by the absence of HER-2 (ErB2) and receptors for estrogen and progesterone [6], by the expression of basal cytokeratins (that is, keratin 5) and proliferation-associated genes [7,8], as well as by defects in genomic gatekeepers, p53, or BRCA1 [9]. While immunohistochemical diagnosis of basal breast cancer is straightforward [6], these patients have limited therapeutic options: the response to mainstay chemotherapy is not uniform and is affected by the type of drugs used [10]; estrogen or HER-2 targeting is not indicated; and attempts to disable ancillary signaling pathways, for instance coordinated by the epidermal growth factor receptor, have so far shown little promise [11]. This adds to a high rate of relapses, which in several series has been linked to shortened overall survival and to death from disease [12].

Although the cell of origin of basal breast cancer has not been conclusively identified [5], a link to the progenitor/stem cell compartment of the mammary epithelium has been proposed [13]. In this context, developmental gene expression pathways that control the interplay between cell proliferation, survival, and differentiation are candidates for stem cell-derived mammary tumorigenesis [14]. One such pathway is centered on the Notch family of cell surface receptors [15] – which affects the mammary stem cell niche [16], and has been associated with malignant transformation [17] and aggressive tumor behavior [18]. Notch expression is correlated to human breast cancer formation but the downstream pathways that guide such behavior are still under investigation [19,20].

Among the candidate effector molecules controlling stem cell viability is survivin, a dual regulator of cell division and apoptosis, broadly overexpressed in cancer [21]. Consistent with its onco-fetal pattern of expression, survivin is essential for tissue homeostasis [21] – and conditional knockout studies have suggested a potential critical role of this pathway in maintaining stem cell viability, at least in certain tissue compartments [22].

In the present study, we used a combination of hierarchical clustering and overall survival analysis of a novel microarray dataset, meta-analysis of published gene profiling studies, and cell culture experiments to investigate a potential role of a Notch-1–survivin signaling axis in breast cancer.

## Materials and methods

### Immunohistochemistry

Nine cases of basal breast cancers with associated clinical and pathological data were obtained from the archives of the Department of Pathology, University of Massachusetts Medical School. Analysis of anonymous discarded tissue with no patient identifiers was approved by and in compliance with Institutional Review Board guidelines.

Tissue sections (5 μm) were cut from paraffin blocks, deparaffinized in xylene, rehydrated, and baked overnight at 60°C. Slides were quenched for endogenous peroxidase with 3% H_2_O_2 _in methanol for 20 minutes, and were processed for antigen retrieval by pressure cooking in 9 mM sodium citrate, pH 6.0, for 20 minutes. Slides were washed in PBS, and incubated overnight at 4°C with a rabbit antibody to the Notch-1 intracellular domain (NIC) or control IgG, were rinsed, and were further incubated with a biotinylated anti-rabbit IgG for 10 minutes at 22°C. After addition of streptavidin-conjugated horseradish peroxidase, the slides were incubated with 3',3'-diamino-benzidine for 3 to 10 minutes, and were counterstained with hematoxylin, as described previously [23].

### Hierarchical clustering analysis of Notch-1 mRNA expression in breast cancer

The log_2 _Cy5/Cy3 ratios of 232 cases of human breast cancer and their associated clinical data were downloaded from the University of North Carolina Microarray Database [24,25]. Only genes where the Lowess normalized intensity values in both channels were > 30 and data existed in > 70% samples were included for analysis. The gene set was further filtered to include only genes with Pearson's correlation coefficient > 0.58 with *Notch-1 *(n = 101).

Two-way hierarchical clustering was performed using Cluster v3 [26], and the results were displayed using JavaTreeview [27]. Analysis of overall survival (log-rank test) was carried out using JMP 6.0 [28] (SAS Institute, Cary, NC, USA) on the subset of breast cancer patients in this cohort with available clinical data (n = 125). Data were plotted for each quartile of normalized *Notch-1 *log_2 _ratios, from highest (first quartile) to lowest (fourth quartile). The breast cancer patients were further divided into basal (n = 35) or nonbasal (n = 88) subgroups and were analyzed for overall survival (log-rank test) using JMP 6.0 [28].

### Meta-analysis of Oncomine microarray data

We reviewed Oncomine [29,30] for independent human breast cancer microarray datasets comparing estrogen receptor (ER)-negative and ER-positive tumors. Databases from 15 studies were found to contain *Notch-1 *and *survivin *relative expression data. The patient characteristics and analyses performed in each study are summarized in Table 1. Descriptive statistics including the mean, standard error, and a two-tailed unpaired *t *test were calculated for the comparisons between ER-positive and ER-negative samples within each study. Separately for ER-negative and ER-positive samples, a Pearson's correlation coefficient (*r*) was calculated for each study to measure levels of pair-wise co-expression between *Notch-1*, *survivin*, and *keratin-5*.

**Table 1:** Published datasets included in the meta-analysis

Study	*n*	Median age (years)	Microarray	Tumor size	Lymph nodes (LN)	Treatment	Stage/grade
Chin and colleagues [55]	118	55.3 (SD = 14.3)	Affymetrix	2.6 cm (SD = 1.3)	67 LN-positive, 51 LN-negative	60% tamoxifen, 52% adjuvant chemotherapy, 51% radiation	26 stage 1, 70 stage 2, 14 stage 3, 5 stage 4; 10 grade 1, 42 grade 2, 61 grade 3; 5 unknown
Desmedt and colleagues [56]	198	47 (all < 61)	Affymetrix	< 5 cm	Node-negative		T1–T2
Ginestier and colleagues [57]	55		Affymetrix			19 amplified for 20q13, 36 unamplified for 20q13	Consecutive cases, unilateral localized invasive breast cancer
Hess and colleagues [58]	133	Training set, 52 (range 29 to 79); validation set, 50 (range 28 to 73)	Affymetrix			Preoperative weekly paclitaxel and fluorouracil–doxorubicin–cyclophosphamide chemotherapy	Stage I, stage II, stage III
Ivshina and colleagues [59]	249 (Uppsala cohort)	62.3	Affymetrix	2.9 cm	35% node-positive	30.3% endocrine therapy, 10.7% chemotherapy, 1.7% combination therapy, 58.8% no systemic therapy	68 grade 1, 126 grade 2, 55 grade 3
Miller and colleagues [60]	251	62.1 (SD = 13.9)	Affymetrix	22.4 mm (SD = 12.5)	84/253 LN metastasis, 160 node-negative, 9 unknown node status	143 no adjuvant therapy; others with systemic adjuvant therapy, and/or chemotherapy	
Minn and colleagues [61]	82	55.8 (SD = 13.5)	Affymetrix	3.68 cm (SD = 1.77 cm)	Average 3.5 (SD = 5.98) axillary LN	Adjuvant chemotherapy and/or hormonal therapy	
Richardson and colleagues [62]	39		Affymetrix				
Saal and colleagues [37]	105	61 (range 26 to 77)	Non-Affymetrix	27 mm (range 2 to 50 mm)	65 (62%) LN-positive	Treated uniformly with 2 years of adjuvant tamoxifen	Stage II, primary breast cancer
Sotiriou and colleagues [63]	119 (KJ125 dataset)	45% < 50, 55% > 50	Affymetrix	61% < 2 cm, 39% > 2 cm	LN-negative	No adjuvant systemic therapy	34 grade 1, 46 grade 2, 28 grade 3, 17 not available
Turashvili and colleagues [32]	10		Affymetrix				3 grade I, 5 grade II, 2 grade III
van de Vijver and colleagues [64]	295	< 52	Non-Affymetrix	< 5 cm	151 LN-negative, 144 LN-positive	Modified radical mastectomy or breast-conserving surgery	Stage I or stage II breast cancer
Wang and colleagues [65]	286	54 (SD = 12)	Affymetrix		LN-negative	No adjuvant treatment	
Yu and colleagues [38]	96 (only 68 with Notch-1 and survivin data)	55 (SD = 10.9)	Affymetrix	37.7 mm (SD = 17.9)	37.5% LN-negative		2 unknown grade, 5 grade I, 26 grade II, 63 grade III
Zhao and colleagues [36]	59 (35 intraductal carcinoma, 17 intralobular carcinoma; three from each with unknown ER status)	Ductal, 53 (SD = 15.5); lobular, 63.5 (SD = 14.0)	Non-Affymetrix		Ductal, 16 LN-positive, 16 LN-negative, 3 LN unknown; lobular, 7 LN-positive, 7 LN-negative, 4 LN unknown		Ductal, 5 grade I, 19 grade II, 11 grade III; lobular, 17 grade II, 1 grade I

Sixteen datasets derived from an unbiased search of human breast cancer microarrays on Oncomine were identified that matched the study criteria.

The 95% confidence interval for *r *was calculated based on Fisher's *Z *transformation [31]. In one study a Fisher's *Z *transformation could not be performed for ER-negative samples (n = 3) [32], and an approximate variance for a Pearson's correlation coefficient was used to derive its 95% confidence interval. To summarize ER-specific results from the individual studies, Fisher's *Z *transformation and its variance were used in pooling correlation from different studies. The weighted average of Fisher's *Z *transformation and its 95% confidence interval were first estimated based on a fixed-effect model, taking into account the variance associated with each study. The ER-specific pooled estimate of Pearson's correlation coefficient and its 95% confidence interval were then derived from the estimates based on the Fisher's *Z *transformation. We applied a random-effect model for meta-analysis [33] to evaluate whether levels of co-expressions between *Notch-1*, *survivin*, and *keratin-5 *differ between ER-negative and ER-positive samples among the different studies.

### Cells, reagents and transfections

The breast adenocarcinoma MDA-MB-231 cell line was obtained from the American Type Culture Collection (Manassas, VA, USA), and was maintained in culture as recommended by the supplier. The cDNA encoding activated NIC was characterized previously [34]. MDA-MB-231 cells were transfected with control plasmid cDNA or NIC cDNA (2 μg) using 6 μl LipofectAmine (Invitrogen, Carlsbad, CA, USA) in Opti-Mem medium (1 ml) (Gibco, Carlsbad, CA, USA). The media was changed after 5 hours, and cells were harvested after 24 hours.

The peptidyl γ-secretase inhibitor z-Leu-Leu-Nle-CHO was purchased from Calbiochem (San Diego, CA, USA), and has been characterized previously [35]. For gene silencing experiments by siRNA, MDA-MB-231 cells were transfected with double-stranded RNA oligonucleotide directed to Notch-1 (pool of three siRNA; Santa Cruz, Santa Cruz, CA, USA), survivin, or control nontargeted sequences using 10 μl HiPerfect (Gibco). Cells under the various conditions were harvested after 48 hours, and were analyzed by western blotting.

## Results

### Expression of Notch-1 and survivin in basal breast cancer

**Figure 1:**
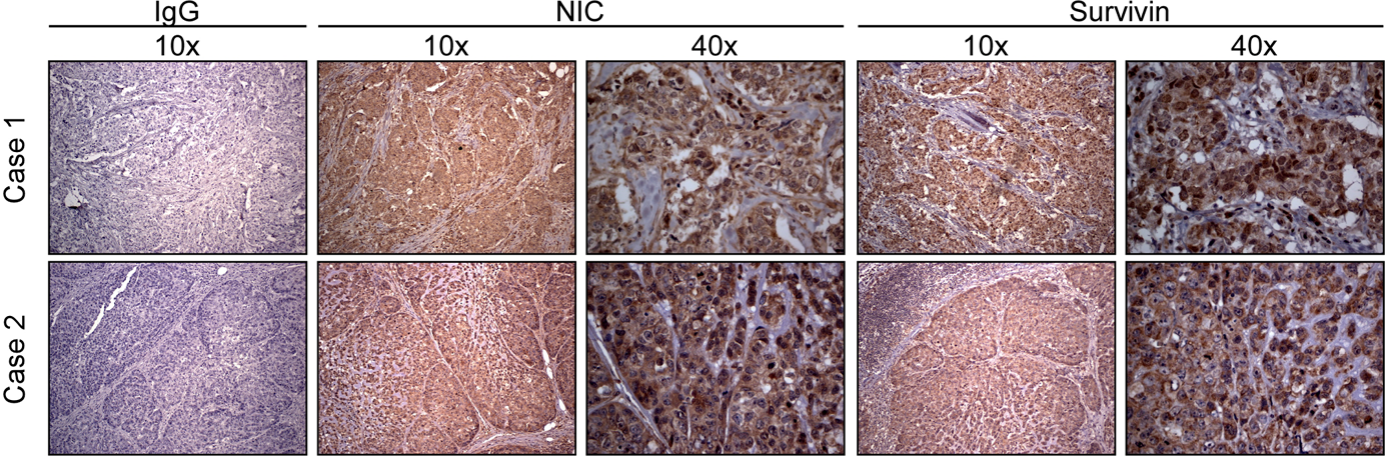
Expression of activated Notch-1 and survivin in basal breast cancer. Representative basal breast cancer cases were analyzed by immunohistochemistry. NIC, Notch-1 intracellular domain.

Recent studies have shown that Notch activation results in increased expression of survivin in basal breast cancer cell lines [35]. To determine whether a similar association occurs *in vivo*, we examined by immunohistochemistry a panel of basal breast cancer cases for expression of activated Notch-1 (NIC) and survivin. The average age of the nine patients was 52.3 ± 6.1 years. All cases were grade 3 tumors with negative protein expression of ER, progesterone receptor, and HER-2, and positive protein expression of keratin 5/6, as assessed by immunohistochemistry. Activated Notch-1 was abundantly expressed in all cases examined of basal breast cancer, and was localized to both the cytosol and nuclei of tumor cells (Figure 1). Survivin was also strongly expressed in all basal breast cancer cases, and was similarly localized to the nuclei and cytosol of the tumor cell population (Figure 1).

### Expression of Notch-1 mRNA in breast cancer microarray databases

**Figure 2:**
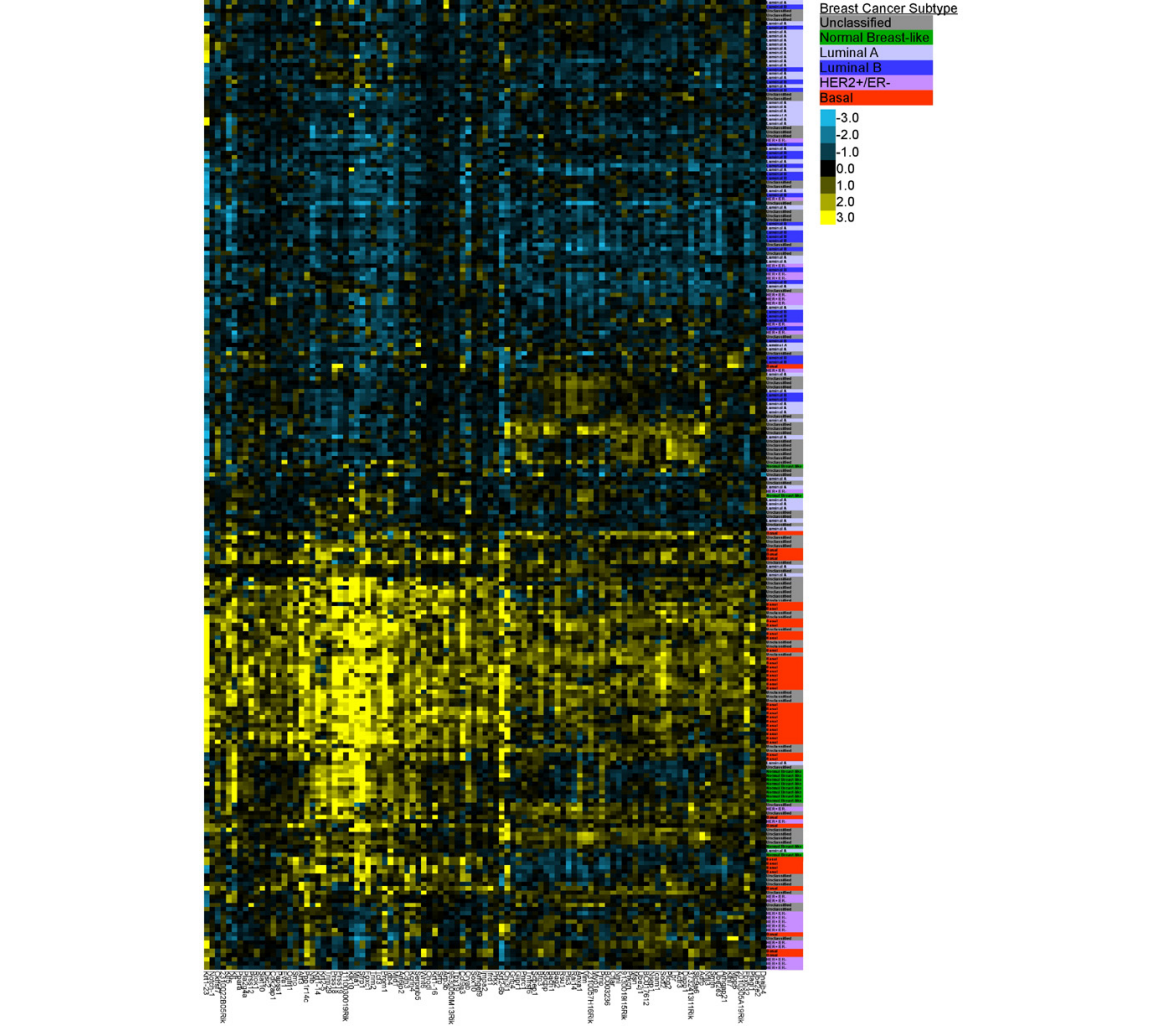
*Notch-1 *segregates with basal breast cancer. Heat map of 232 cases of breast cancer. Unclassified, gray; normal breast-like, green; luminal A, light blue; luminal B, dark blue; HER-2+/estrogen receptor-negative (ER-), purple; basal, red.

We next analyzed the expression of Notch-1 mRNA in an established breast cancer patient cohort. Supervised hierarchical clustering of 232 cases of human breast cancer [25], using intrinsic gene analysis, revealed that higher expression of *Notch-1 *segregated with basal breast cancer. Other known markers of the disease, including *keratin-5*, *keratin-14*, and *kit*, were also highly correlated with *Notch-1 *expression (*r *≥ 0.58) in this cohort (Figure 2).

**Figure 3:**
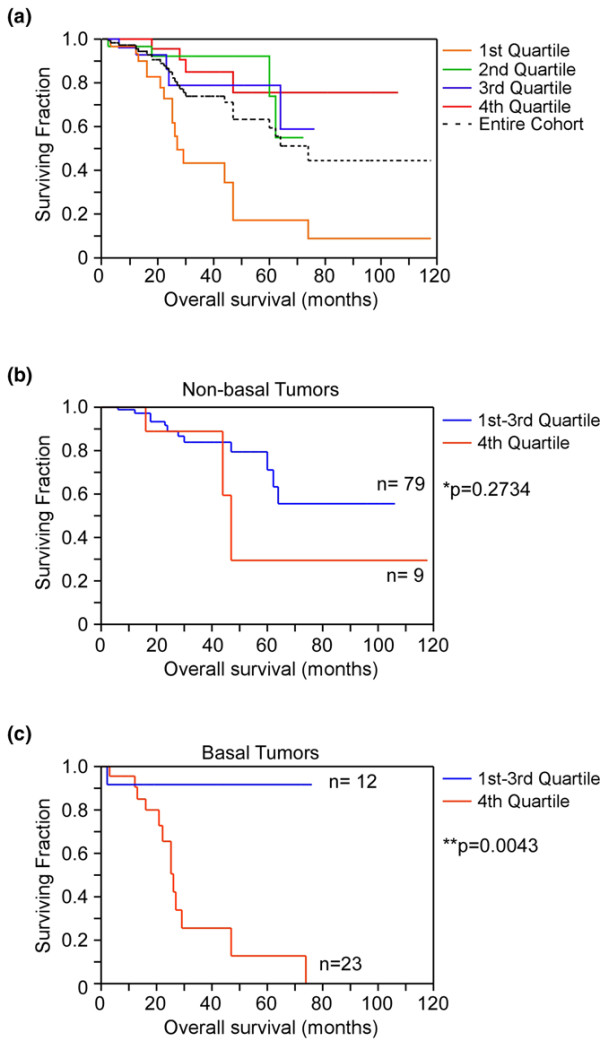
Overall survival. **(a) **Kaplan–Meier curves of differential overall survival were plotted according to Notch-1 expression in the entire cohort (quartiles). **(b) **Nonbasal tumors and **(c) **basal tumors were subgrouped into high Notch-1 expression (fourth quartile) or low Notch-1 expression (first to third quartiles) and were analyzed for overall survival.

One hundred and twenty-five patients with associated clinical outcome data were further analyzed. When stratified according to levels of *Notch-1 *log_2 _transcript ratios, tumors with the highest quartile of *Notch-1 *gene expression (first quartile) exhibited abbreviated overall survival with a median survival of 27 months compared with the other groups (*P *< 0.001 via the log-rank test) (Figure 3a). Seventy-two percent of tumors in this first quartile (23/32 tumors) were classified as basal breast cancer, and the overall survival of these patients was approximately 50% lower of that of the remaining population (*P *< 0.02). Conversely, reduced levels of *Notch-1 *(second to fourth quartiles) were associated with better overall survival (Figure 3a). The percentage of basal breast cancers in these groups was 17% (second quartile, 5/30 tumors), 20% (third quartile, 6/30 tumors), and 10% (fourth quartile, 3/31 tumors), respectively.

The tumors were then segregated based on basal or nonbasal subgroup status and were analyzed for overall survival. In the nonbasal breast tumors, the expression of *Notch-1 *was not associated with significant differences in overall survival (*P *= 0.2734) (Figure 3b). In contrast, *Notch-1 *levels dictated overall survival in basal breast tumors (Figure 3c). In this basal subgroup, increased *Notch-1 *expression (fourth quartile) greatly reduced overall survival to the median of 26 months (Figure 3c). Comparatively, low levels of *Notch-1 *(first to third quartiles) demonstrated improved overall survival (Figure 3c).

### Gene expression correlation in basal breast cancer

We next carried out a meta-analysis of published microarray datasets to identify genes associated with Notch and potentially implicated in the molecular pathogenesis of basal breast cancer. Based on our recent data [35], we focused on *survivin *– a mitotic regulator and cell death inhibitor overexpressed in breast cancer [3,21], and associated with unfavorable outcome [2] – and *keratin-5 *– a marker of basal epithelium, often linked to a progenitor/stem cell phenotype [13].

Fifteen microarray datasets, mostly employing Affymetrix technology, published between 2002 and 2007 met the search criteria (Table 1). The overall median age of patients was 55.2 years. The breast tumors examined were typically < 5 cm, encompassing all grades, and included lymph node-positive and lymph node-negative disease. In one study, separate databases for lobular and ductal breast cancer were examined [36] – bringing the datasets analyzed to a total of 16. Two studies did not contain downloadable *keratin-5 *expression data [36,37], and one study contained 68 out of 96 samples with *Notch-1 *and *survivin *data and 19 samples with *keratin-5 *data [38]. Table 2 presents the descriptive statistics of each cohort with respect to *Notch-1*, *survivin*, and *keratin-5 *relative expression.

**Table 2:** Descriptive statistics of studies in the meta-analysis

Study	Total *n*	Subset	Survivin			Notch-1			Keratin 5		
					
			Mean	SEM	*P*	Mean	SEM	*P*	Mean	SEM	*P*
Chin and colleagues [55]	118	ER- (n = 43)	0.7723	0.0815	1.353 × 10^-5^	0.7793	0.0548	2.231 × 10^-5^	0.9844	0.1436	4.893 × 10^-6^
		ER+ (n = 75)	0.3371	0.0437		0.5027	0.0245		0.1884	0.0678	

Desmedt and colleagues [56]	198	ER- (n = 64)	0.7238	0.0478	1.414 × 10^-8^	0.3254	0.0389	3.951 × 10^-4^	0.6649	0.0996	1.159 × 10^-2^
		ER+ (n = 134)	0.3278	0.0460		0.1658	0.0193		0.3773	0.0507	

Ginestier and colleagues [57]	55	ER- (n = 28)	0.7621	0.1251	9.689 × 10^-5^	1.4343	0.0504	2.933 × 10^-4^	1.2587	0.2116	1.219 × 10^-2^
		ER+ (n = 27)	0.4959	0.0954		1.1536	0.0520		0.5842	0.1492	

Hess and colleagues [58]	133	ER- (n = 51)	0.0776	0.0451	2.071 × 10^-3^	0.2169	0.0454	1.203 × 10^-1^	0.5110	0.1706	1.165 × 10^-4^
		ER+ (n = 82)	-0.0956	0.0309		0.1238	0.0384		-0.2216	0.0480	

Ivshina and colleagues [59]	249^a^	ER- (n = 34)	0.4877	0.0847	1.064e × 10^-7^	0.3819	0.0589	1.643 × 10^-2^	0.7071	0.1683	1.403 × 10^-1^
		ER+ (n = 211)	-0.0950	0.0408		0.2299	0.0137		0.4438	0.0479	

Miller and colleagues [60]	251	ER- (n = 34)	0.3701	0.0681	2.780 × 10^-8^	0.3011	0.0443	2.073 × 10^-2^	0.4714	0.1310	2.638 × 10^-1^
		ER+ (n = 213)	-0.1337	0.0382		0.1905	0.0113		0.3165	0.0389	

Minn and colleagues [61]	82	ER- (n = 42)	0.1969	0.0719	1.809 × 10^-4^	0.4515	0.0529	7.897 × 10^-5^	1.1060	0.1311	2.391 × 10^-6^
		ER+ (n = 57)	-0.1821	0.0652		0.2036	0.0247		0.2624	0.1028	

Richardson and colleagues [62]	39	ER- (n = 24)	-0.1141	0.1363	6.374 × 10^-3^	1.1360	0.0621	1.216 × 10^-1^	1.1878	0.1436	4.670e × 10^-3^
		ER+ (n = 15)	-0.6187	0.1089		1.0139	0.0457		0.6193	0.1225	

Saal and colleagues [37]	105	ER- (n = 60)	-1.3798	0.1075	5.143 × 10^-6^	0.2043	0.0756	3.033 × 10^-3^			
		ER+ (n = 45)	-2.1290	0.1121		-0.1145	0.0728				

Sotiriou and colleagues [63]	119	ER- (n = 34)	0.4834	0.0320	2.528 × 10^-3^	0.2702	0.0565	1.323 × 10^-4^	0.9849	0.1107	9.396 × 10^-3^
		ER+ (n = 85)	0.3691	0.0166		0.0181	0.0196		0.6560	0.0499	

Turashvili [32]	10	ER- (n = 3)	0.1268	0.1688	2.533 × 10^-1^	1.1991	0.1243	3.082 × 10^-1^	0.6117	0.3735	6.750 × 10^-1^
		ER+ (n = 7)	-0.2686	0.2733		1.0239	0.0709		0.3655	0.4358	

van de Vijver and colleagues [64]	295	ER- (n = 69)	0.3719	0.2218	1.847 × 10^-12^	0.6277	0.1148	1.418 × 10^-10^	-0.7917	0.4073	1.748 × 10^-5^
		ER+ (n = 226)	-1.7301	0.1580		-0.2900	0.0590		-2.8083	0.1822	

Wang and colleagues [65]	286	ER- (n = 77)	0.0902	0.0459	2.284 × 10^-8^	0.4139	0.0282	2.255 × 10^-11^	0.8906	0.0819	3.242 × 10^-7^
		ER+ (n = 209)	-0.2387	0.0318		0.1784	0.0151		0.3844	0.0459	

Yu and colleagues [38]	68 (19)	ER- (n = 15)	-0.2546	0.0874	1.689 × 10^-1^	-0.2712	0.1090	1.946 × 10^-4^	1.8394*	0.2312*	2.280 × 10^-2 ^*
		ER+ (n = 4)	-0.4225	0.0831		0.7625	0.0431		0.9196*	0.2431*	

Zhao and colleagues (lobular) [36]	16	ER- (n = 4)	-2.4418	0.2721	6.851 × 10^-1^	0.0416	0.4079	7.011 × 10^-1^			
		ER+ (n = 12)	-2.3108	0.1269		-0.1367	0.1083				

Zhao and colleagues (ductal) [36]	34	ER- (n = 11)	-1.4613	0.2938	1.440 × 10^-1^	0.3298	0.3722	1.769 × 10^-1^			
		ER+ (n = 23)	-2.0013	0.1981		-0.2272	0.1104				

Mean, standard error of mean (SEM), and *P *value between estrogen receptor-negative (ER-) and estrogen receptor-positive (ER+) breast cancers are presented for *Notch-1*, *survivin*, and *keratin-5 *expression in the analyzed datasets. ^a^Uppsala cohort. * Subset of samples with *keratin-5 *data.

### A novel dual-gene signature in basal breast cancer

**Figure 4:**
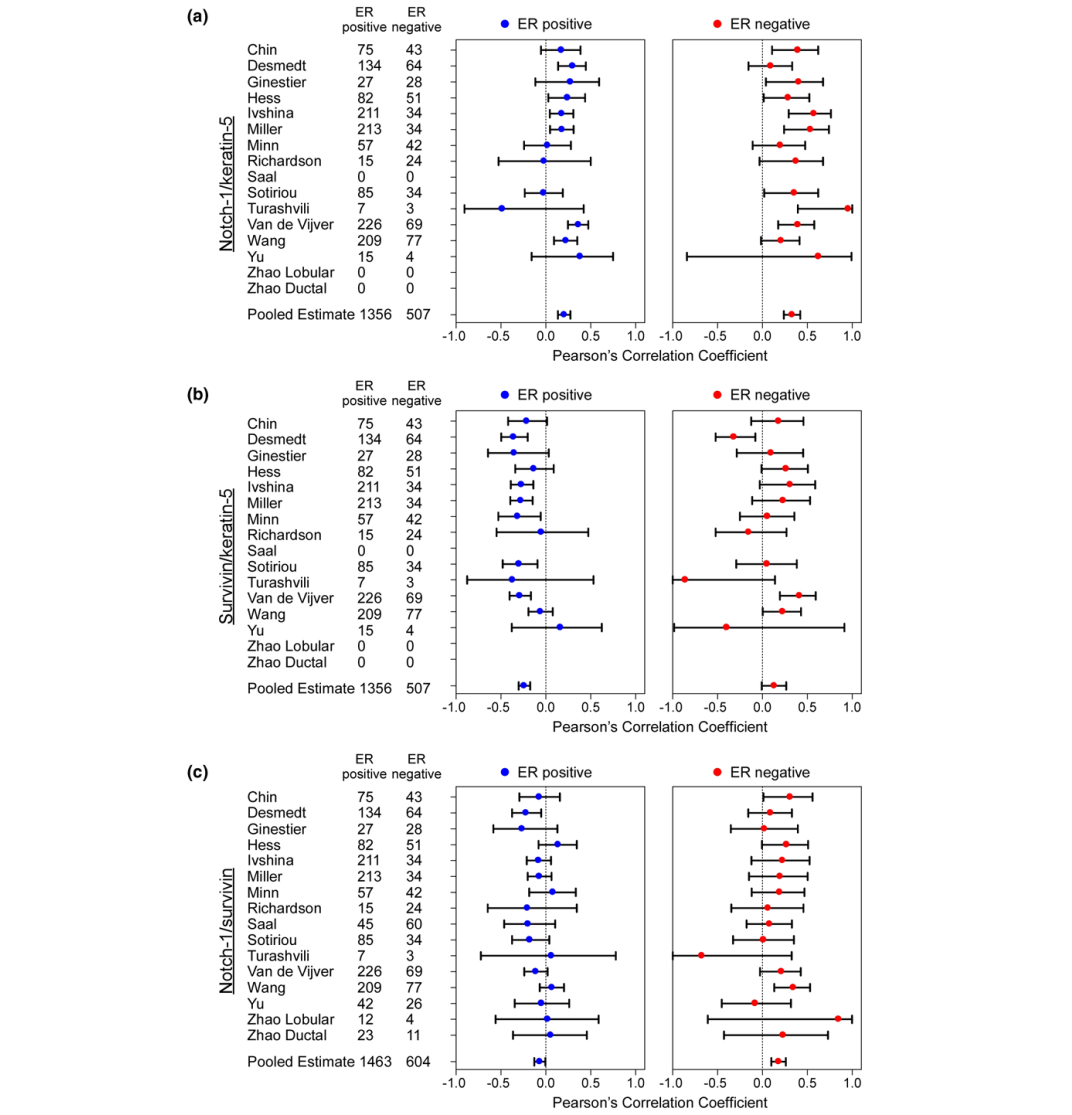
Co-segregation of *Notch-1*, *survivin*, and *keratin-5 *in breast cancer. Pearson's correlation coefficient and the 95% confidence interval were calculated from the analysis of individual datasets. **(a) ***Notch-1*/*keratin-5*. **(b) ***Survivin*/*keratin-5*. **(c) ***Notch-1/survivin*. For study details, see Table 2. ER, estrogen receptor.

Analysis of 507 ER-negative and 1,356 ER-positive breast cancer patients revealed that *keratin-5 *associated with ER-negative breast cancers (Figure 4a) in seven out of 13 datasets, and that *Notch-1 *associated with ER-negative breast cancers in nine out of 16 datasets (Table 2). Pooled estimates of Pearson's correlation coefficient between *Notch-1*/*keratin-5 *were 0.3315 and 0.2043 for ER-negative and ER-positive breast cancers, respectively (*P *= 0.04) (Figure 4a). Similarly, *survivin *and *keratin-5 *co-segregated in ER-negative breast cancer, with a pooled estimate of Pearson's correlation coefficient of 0.1314 for ER-negative breast cancer and of -0.2408 for ER-positive breast cancer (*P *< 0.0001) (Figure 4b). A negative correlation exists between *survivin *and *keratin-5 *in ER-positive breast cancers most probably because other transcriptional and nontranscriptional mechanisms are likely to control survivin expression in nonbasal cancers (Figure 4b).

Analysis of 604 ER-negative and 1,463 ER-positive breast cancer patients revealed that *survivin *segregated with ER-negative tumors (two-tailed *P *< 0.05) in 12 out of 16 cohorts (Table 2). The Pearson's correlation coefficients between *Notch-1 *and *survivin *were 0.1804 and -0.0674 for ER-negative and ER-positive breast cancers, respectively (*P *< 0.0001) (Figure 4c).

### Notch-1 regulation of survivin expression

Consistent with the model presented above, recent studies have shown that survivin may function as a direct transcriptional target of Notch-1, thus controlling mitotic transition and resistance to apoptosis in breast cancer [35]. In agreement with these data, transfection of ER-negative breast cancer MDA-MB-231 cells with NIC resulted in increased survivin expression, as determined by western blotting, whereas acute siRNA silencing of Notch was associated with reduced survivin levels and induction of apoptosis (data not shown). Similarly, inhibition of Notch signaling by a pharmacologic inhibitor of γ-secretase suppressed *survivin *gene expression (data not shown), validating the identity of survivin as a direct transcriptional target of Notch in breast cancer cells [35].

## Discussion

In the present study, we have shown that Notch-1 is preferentially expressed in breast cancer, as compared with normal tissues, segregates with basal disease, and correlates with abbreviated survival. In a meta-analysis of multiple, independent microarray datasets, *Notch-1*, *survivin*, and *keratin-5 *selectively co-associated with ER-negative versus ER-positive breast cancer patients. Consistent with recent observations [35], survivin was validated as a direct transcriptional target of Notch in model ER-negative breast cancer cells.

These findings add to an in-depth molecular classification of breast cancer [4] – and in particular basal breast cancer, a disease variant that still poses significant therapeutic challenges. In addition to high-risk genetics [7,8] and aggressive histologic features [5], it has been speculated that basal breast cancer may originate from a progenitor/stem cell compartment in the basal mammary epithelium. This is consistent with a proposed role for Notch in mammary progenitor cell differentiation and maintenance [39], and potentially in the early events of their transformation [40]. Such a pathway may not be exclusively limited to breast cancer [20], given that deregulated Notch signaling has been implicated as a driver of disparate malignancies [15], as promoting aberrant cell cycle progression [41], and associated with unfavorable outcome [18].

In this context, survivin appears ideally suited to function as a pleiotropic, *direct *Notch effector gene in clinically aggressive breast cancer [2]. At the molecular level, this involves occupancy of discrete RPB-Jκ binding element(s) in the *survivin *promoter upon Notch activation, which results in transcriptional upregulation of survivin levels, inhibition of apoptosis, and acceleration of mitotic transitions selectively in ER-negative breast cancer cells [35]. Whether deregulation of a Notch–survivin signaling axis is preferentially operative in a progenitor/stem cell compartment is currently not known.

Intriguing, however, is that another developmental gene expression pathway (that is, Wnt/β-catenin) has been implicated in controlling survivin levels in intestinal crypt progenitor cells, potentially contributing to colon cancer [42], and that survivin expression been consistently associated with stemness gene signatures of mesenchymal [43], neuronal [44], and skin [45] progenitor cells. Results of conditional knockout studies appear to support this model, as heterozygous deletion of survivin produced complete bone marrow ablation, loss of hematopoietic progenitor/stem cells, and rapid animal mortality [22]. This pathway may have a clear link to human disease, as lineage-specific methylation and silencing of the *survivin *gene has been linked to bone marrow depletion in myelodsyplastic syndrome [46]. With respect to breast cancer, Notch-dependent upregulation of survivin [35] may broadly suppress apoptosis, deregulate cell cycle progression [21], and ultimately promote resistance to mainstay therapeutic agents in this disease, such as taxanes [47] and DNA damaging agents [48].

Although the diagnosis of triple-negative, basal breast cancer is straightforward [6], these patients continue to pose therapeutic challenges for the aggressive nature of the disease, which is prone to relapse, and the lack of appropriate, molecularly targeted agents [10]. Based on the findings presented herein, it may be possible to envision antagonists of Notch [49] and of survivin [21] as potential molecular therapy for basal breast cancer patients. Agents that interfere with Notch signaling inhibit the enzyme γ-secretase, which is responsible for the activating intracellular cleavage of Notch upon ligand binding at the cell surface [15]. Despite concerns of specificity [50] and potential intestinal toxicity [51], γ-secretase inhibitor molecules are being tested as molecular therapy for leukemic patients harboring activating mutations in Notch [49]. In our recent studies, systemic administration of a peptidyl γ-secretase inhibitor significantly inhibited breast cancer growth *in vivo*, and almost completely abolished metastatic dissemination, with no detectable organ or systemic toxicity [35]. Antagonists of survivin are also available in the clinic, producing encouraging patient responses and manageable toxicity in early-phase clinical trials [21].

In summary, we have extended recent *in vitro *observations [35] and have validated the existence of a functional Notch-1/survivin signaling axis, *in vivo*, selectively in patients with basal breast cancer. Targeting Notch-1 signaling in model breast cancer cells lowered survivin levels, resulting in pronounced anti-tumor effects [35]. Taken together with the stringent correlation reported here across disparate tumor series, *in vivo*, this observation raises the possibility that basal breast cancer cells may selectively become dependent on, or addicted to, Notch/survivin signaling for their maintenance [52]. Although it is unclear to what extent oncogene addiction maintains the malignant phenotype *in vivo *[53], antagonists of such pathways have produced impressive clinical responses, at least in certain patient subsets [54]. A similar rationale may be envisioned here for targeting Notch and survivin in basal breast cancer patients, especially if this pathway can be disabled in a progenitor/stem cell compartment, acting as a potential disease reservoir contributing to a high incidence of relapses.

## Conclusion

Expression of Notch-1 and survivin segregates with clinically aggressive and recurrence-prone basal breast cancer. Antagonists of these signaling pathways may be considered as targeted, novel molecular therapy of basal breast cancer.

## Abbreviations

ER: estrogen receptor; HER-2: human epidermal growth factor receptor 2; NIC: Notch-1 intracellular domain; PBS: phosphate-buffered saline; siRNA: small interfering RNA.

## Competing interests

The authors declare that they have no competing interests.

## Authors' contributions

CWL, JP, and MG carried out experiments of survivin and Notch expression and function in breast cancer as well as immunohistochemical analysis in primary normal and tumor samples. KS performed the hierarchical clustering experiments. QL and C-CH provided statistical evaluation and interpretation of the microarray and Pearson analysis data. AK analyzed the immunohistochemistry of human breast tumor samples and provided clinical and pathological data. DCA participated in the design and coordination of the study. CWL and DCA wrote the paper. All authors read and approved the final manuscript.
